# Relationship Between Vertigo and Consumption of Psychotropic Drugs: A Prospective Case–Control Study

**DOI:** 10.3390/jcm14082555

**Published:** 2025-04-08

**Authors:** Inés Sánchez-Sellero, Andrés Soto-Varela

**Affiliations:** 1 Division of Toxicology, Department of Forensic Sciences, Pathology, Gynecology and Obstetrics, and Pediatrics, School of Medicine, Universidade de Santiago de Compostela, 15782 Santiago de Compostela, Spain; ines.sanchez.sellero@usc.es; 2 Forensic Toxicology Service, Forensic Sciences Institute, Universidade de Santiago de Compostela, 15782 Santiago de Compostela, Spain; 3 Division of Neurotology, Department of Otorhinolaryngology, Complexo Hospitalario Universitario de Santiago de Compostela, 15706 Santiago de Compostela, Spain; 4 Department of Surgery and Medical-Surgical Specialities, School of Medicine, Universidade de Santiago de Compostela, 15782 Santiago de Compostela, Spain; 5 Health Research Institute of Santiago (IDIS), 15706 Santiago de Compostela, Spain

**Keywords:** vertigo, psychotropic drug, psychological distress, anxiety, depression, quality of life

## Abstract

**Background/Objectives**: The association between vestibular symptoms and psychological distress has been previously studied, mainly with the use of questionnaires. The purpose of this study is to compare the consumption of psychotropic drugs between a group of patients with vertigo and a control group. **Methods**: A prospective cross-sectional, observational, case–control study was carried out, including 506 patients (232 with Ménière’s disease, 79 with vestibular migraine, 34 with vestibular neuritis, and 161 with benign paroxysmal positional vertigo). In total, 253 participants were included in the control group. Both groups were comparable regarding age, sex, and history of previous psychiatric diseases. **Results**: The percentage of patients with vertigo who consumed psychotropic drugs (41.3%) was higher than the percentage of the control group who did so (26.9%) (Fisher’s exact test, *p* < 0.0001; OR = 1.914, CI95% (1.377; 2.662)). The mean number of psychotropic drugs consumed was also higher (Mann–Whitney test, *p* = 0.0003) in cases (0.68 ± 0.959) than in controls (0.47 ± 0.889). This higher consumption in the group of patients with vertigo was found for all pharmacological groups studied, being especially relevant regarding “anxiolytics and hypnotics and sedatives” and “antidepressants”. No statistically significant differences in the consumption of psychotropic drugs between types of vestibular disorders were observed. The longer the symptoms were present, the higher the prevalence of psychotropic drug use was observed. **Conclusions**: A relationship between vertigo and consumption of psychotropic drugs was found. Recording the consumption of these drugs is proposed as an objective method to better understand the psychological distress that patients with vertigo may suffer from.

## 1. Introduction

Vertigo is a disabling symptom that can severely impact daily activities and greatly diminish an individual’s quality of life [1,2]. It was reported that the presence of continuous instability might cause severe handicapping effects in patients suffering from advanced stages of Ménière’s disease, in bilateral cases of this disease or as a side effect of destructive procedures [1]. On the other hand, it was also reported that benign paroxysmal positional vertigo (BPPV) significantly impacts the quality of life, despite the short duration of episodes of vertigo [2]. Especially in patients with episodic vertigo, the recurrence and, in many cases, the unpredictable nature of vertigo episodes can be emotionally distressing and cause limitations and restrictions in activities and professional and social participation. All these effects can have a profound impact on quality of life [2]. The physical functioning of patients with definite vestibular migraine is highly affected by their dizziness, resulting in a lower quality of life, compared to patients with migraine without vertigo and healthy people [3].

Whether vertigo originates from a central or peripheral cause, it is often accompanied by psychiatric symptoms. Patients suffering from long-term vertigo syndrome often experience emotional distress and mood affectation, which sometimes lead to the development of psychopathological conditions [3,4,5,6]. High levels of prescription drug usage, including psychotropic medication, have been reported in elderly people with dizziness [7]. A recent systematic review showed that 9% of elderly patients with vertigo had a psychiatric diagnosis, such as depressive syndrome and anxiety. These conditions can increase the fear of vertigo attacks and consequently the fear of falling, limiting daily activities and establishing a vicious circle [8].

Mental disorders such as anxiety and/or depression may overlap with vestibular symptoms, strengthening them and thus generating a vicious circle that considerably worsens the quality of life of patients, reduces daily activities, and even limits the ability to work. The relationship between anxiety and vertigo is complex. Anxiety can cause vertigo (a symptom in panic attacks), but anxiety is often a secondary complication of vertigo. Diagnosis of anxiety disorders was less frequent in patients with bilateral chronic vestibular disorders than in those with episodic vertigo/dizziness, although the levels of anxiety were found to be similar between both groups [9].

This association between vestibular symptoms and mental health disorders or psychological distress has already been studied [3,4,5,6,10]. In patients suffering from Ménière’s disease, it has been reported that unusual events and stress may act as attack triggers and exacerbate symptoms [10], and these effects can produce and increase stress. In patients with Ménière’s disease and vestibular migraine, a higher prevalence of psychiatric comorbidities, mainly anxiety and depressive disorders, was reported [11].

The systematic review about vertigo in the elderly previously mentioned proposed polypharmacy as a possible concurrent source of vertigo in these patients. In a variable percentage of elderly patients under this condition (i.e., prescription of more than three different types of drugs), the occurrence of vertigo was increased. Antihypertensive and psychotropic drugs, especially sedative–hypnotic drugs, were the main involved medications, commonly administered as chronic treatment [8].

The treatment of vestibular disorders often includes the prescription of psychotropic drugs. To manage Ménière’s disease symptoms in the acute phase, standard pharmacological treatments are anti-vertigo drugs or vestibular suppressants, including antihistamines, anticholinergics, and benzodiazepines [12]. GABA is an inhibitory neurotransmitter in the vestibular system. Benzodiazepines act as a positive allosteric modulator of GABA-A receptor and cause inhibitory action in the central nervous system. This inhibitory action of benzodiazepines also shows therapeutic effects in mood disorders commonly associated with acute vertigo in Ménière’s disease, such as depression and anxiety [12,13]. Regarding BPPV, the update group of a clinical practice guideline from the American Academy of Otolaryngology–Head and Neck Surgery Foundation made recommendations against routinely treating BPPV with vestibular suppressant medications such as antihistamines and/or benzodiazepines [14]. The management of vestibular migraine includes preventive and/or rescue medications. Pharmacological management includes anticonvulsants, especially lamotrigine if vertigo is more frequent than headaches, and antidepressants. Tricyclic antidepressants such as amitryptiline or nortryptiline are used in patients with comorbid sleep disturbance or comorbid depression or anxiety [15,16,17]. If psychiatric symptoms are prominent, benzodiazepines and serotonin reuptake inhibitors are usually prescribed [15]. The pharmacological management of vestibular neuritis includes vestibular suppressants. Antihistamines, benzodiazepines, and anticholinergics are options in the first 2−3 days. Therefore, vestibular suppressants are widely used in the pharmacological treatment of acute vestibular syndrome [18].

Emotional and psychological effects and mental health were assessed mainly with the use of questionnaires such as the Hospital Anxiety and Depression Scale (HADS), Dizziness Handicap Inventory (DHI), and 36-Item Short Form Health Survey (SF-36), which allow the subjective assessment of the level of anxiety, depression, or emotional distress in patients with vertigo. DHI and SF-36 are tools used to evaluate the impact of vertigo on quality of life. DHI includes items subgrouped into three domains, namely physical, emotional, and functional, and evaluates self-perceived handicaps in everyday life. SF-36 generates scores across eight dimensions of health: physical functioning, general health, role physical, bodily pain, social functioning, vitality, role emotional, and mental health. These self-reported questionnaires are subjective.

A relatively objective method of assessing the mental health status of patients with vertigo is to record the psychotropic drugs consumed. While questionnaires assess the subjective perception of the patient, prescribing drugs requires an evaluation by a physician who has recognized the reported symptoms as severe enough to require pharmacological treatment.

Therefore, the aim of this study is to compare the consumption of psychotropic drugs between a group of patients with vertigo and a control group of individuals with otorhinolaryngological disorders that do not affect the vestibular system. We hypothesize that patients with vertigo consume more psychotropic drugs than controls.

## 2. Materials and Methods

### 2.1. Design

This is a prospective cross-sectional, observational, case–control study. Data were prospectively collected and recorded for three years (2021−2023). Participants were recruited from patients suffering from vertigo attended in a Neurotology Unit of a tertiary referral hospital.

### 2.2. Inclusion Criterion

The inclusion criterion was patients affected by vertigo, with a definite diagnosis of any of the following four disorders:

Definite Ménière’s disease (MD), according to the Classification Committee of the Bárány Society [19]:

- Two or more spontaneous episodes of vertigo, each lasting 20 min to 12 h.
- Audiometrically documented low- to medium-frequency sensorineural hearing loss in one ear, defining the affected ear on at least one occasion before, during, or after one of the episodes of vertigo.
- Fluctuating aural symptoms (hearing, tinnitus, or fullness) in the affected ear.
- Not better accounted for by another vestibular diagnosis.

Definite Vestibular Migraine (VM), according to diagnostic criteria formulated by the Committee for Classification of Vestibular Disorders of the Bárány Society, published in 2012 [20] and revised in 2022 [21]:

- At least 5 episodes with vestibular symptoms of moderate or severe intensity, lasting 5 min to 72 h.
- Current or previous history of migraine with or without aura according to the International Classification of Headache Disorders (ICHD-3).
- One or more migraine features with at least 50% of the vestibular episodes:
  ○ Headache with at least two of the following characteristics: one-sided location, pulsating quality, moderate or severe pain intensity, aggravation by routine physical activity.
  ○ Photophobia and phonophobia.
  ○ Visual aura.
- Not better accounted for by another vestibular or ICHD diagnosis.

Vestibular neuritis (VN) (acute vestibular syndrome, with objective data of unilateral vestibular damage, without hearing affectation and without data of injury to the central nervous system).

BPPV, according to Bárány Society criteria [22]:

- Recurrent attacks of positional vertigo or positional dizziness provoked by lying down or turning over in the supine position.
- Duration of attacks < 1 min.
- Positional nystagmus elicited after a latency of one or few seconds by the Dix–Hallpike maneuver, the side-lying maneuver (Semont diagnostic maneuver), or the supine roll test.
- Not attributable to another disorder.

### 2.3. Exclusion Criteria

Patients who met the following criteria were excluded:

- Age < 18 years old;
- Simultaneous diagnosis of two or more types of vertigo;
- Other causes of vestibular symptoms (including other central vestibular disorders besides VM) different from those mentioned above in the *Inclusion Criterion* section;
- Severe cognitive impairment that prevents providing informed consent.

### 2.4. Control Group

A total of 253 patients were included in the control group. These patients were recruited from the Otorhinolaryngology Service where they had attended for different reasons, without current or past vestibular symptoms. A case–control ratio of 2:1 was established. Individuals included in the control group were comparable in sex and age with the patients included in the vertigo sample.

### 2.5. Methodology

To establish the diagnosis, all patients affected by vertigo underwent a complete neuro-otological assessment. This included otoscopy, basic neurological exploration, observation and recording of spontaneous nystagmus, video-assisted head impulse test (vHIT, using the ICS Impulse^®^ of Otometrics, Natus Medical, Middelton, WI, USA), positional tests (Dix and Hallpike, McClure, and cephalic hyperextension tests), and pure tone audiometry (using the Interacoustics Audiotest model 340 audiometer, Interacoustics, Middelfart, Denmark). When necessary, caloric tests and vestibular-evoked myogenic potential tests were also performed. All patients diagnosed with MD underwent encephalic MRI to exclude other possible causes of their symptoms.

The following variables were collected and analyzed:

- Age at the time of this study;
- Sex;
- Age at onset;
- The subtypes of BPPV and their locations (side and canal affected);
- The side distribution in MD;
- The side and the branch of the vestibular nerve affected in VN;
- History of psychiatric diseases;
- Psychotropic drugs regularly consumed (≥1 month, while sporadic or unscheduled consumption was excluded), by reviewing the electronic medical record and directly asking the participants about their consumption. The number of different drugs consumed by each patient was recorded. Drugs were grouped into the following categories, applying the Anatomical, Therapeutic, Chemical Classification System (ATC code):
  - Anxiolytics (N05B) (e.g., diazepam, alprazolam, bromazepam, lorazepam, clorazepate, ketazolam) and hypnotics and sedatives (N05C) group (e.g., lormetazepam, zolpidem);
  - Antidepressant (N06A) group (e.g., amitriptyline, nortriptyline, clomipramine, fluoxetine, sertraline, paroxetine, fluvoxamine, escitalopram, venlafaxine, desvenlafaxine, mirtazapine, trazodone, duloxetine, reboxetine);
  - Antiepileptic (N03A) group (e.g., pregabalin, gabapentin, sodium valproate);
  - Antipsychotic (N05A) group (e.g., quetiapine, olanzapine, clozapine, aripiprazole, paliperidone, tiapride).

Combinations of the above drugs also were analyzed.

### 2.6. Sample Size Estimation

For the sample size estimation, we used the percentage of subjects who consumed psychotropic drugs as a reference, which was 26.9% in the control group. We estimated a difference between both groups of 10 percentage points to be significant. With a 95% confidence level (1-α) and 90% statistical power, for a bilateral hypothesis test, a total of at least 455 subjects were estimated to be necessary. Therefore, a total of 506 patients were included (to maintain the 2:1 ratio with the control group), considered enough to obtain statistically significant conclusions.

### 2.7. Sample

As mentioned above, the sample consisted of 506 patients (232 with MD, 79 with VM, 34 with VN, and 161 with BPPV). The mean age of this group was 58.8 ± 14.764 years, and the female/male ratio was 2.1:1 (341 women and 165 men). Regarding the control group, 253 participants were included, showing a mean age of 56.7 ± 15.911 years and a female/male ratio of 1.7:1 (160 women and 93 men). Both groups (cases and controls) were comparable regarding age (*p* = 0.128, Mann–Whitney test) and sex (*p* = 0.257, Fisher’s exact test).

### 2.8. Statistical Analysis

Data were collected and incorporated into a database created for this purpose. Statistical analysis of results was performed with the SPSS version 15.0 for Windows software program (IBM, Chicago, IL, USA). The Kolmogorov–Smirnov test was applied to assess if continuous variables showed a normal distribution. When normality could be assumed, relationships between those variables and the discrete variables were evaluated by the t-Student test. When continuous variables did not show a normal distribution, the Mann–Whitney non-parametric test was applied to analyze the relationships. The Chi-square test was applied to analyze relationships between discrete variables, Fisher’s exact test was used to analyze 2 × 2 tables, and the results are presented as odds ratios with 95% confidence intervals. The statistical significance level in all tests applied was considered as *p* value < 0.05.

### 2.9. Ethical Considerations

This study was reviewed and approved by the local Independent Ethics Committee (Santiago-Lugo Research Ethics Committee, Santiago de Compostela, Spain), protocol code 2018/527 (approval date: 19 December 2018), and was conducted according to the Declaration of Helsinki. All participants provided informed consent.

## 3. Results

In total, 232 patients with MD (139 women and 93 men), 79 patients affected by VM (60 women and 19 men), 34 with VN (24 women and 10 men), and 161 with BPPV (118 women and 43 men) were included in the study group. The general characteristics of the study population can be observed in Table 1. The history of previous psychiatric diseases was analyzed, and no statistically significant differences were observed between cases (20.55%) and controls (25.98%) (*p* = 0.242, Fisher’s exact test).

**Figure 1:**
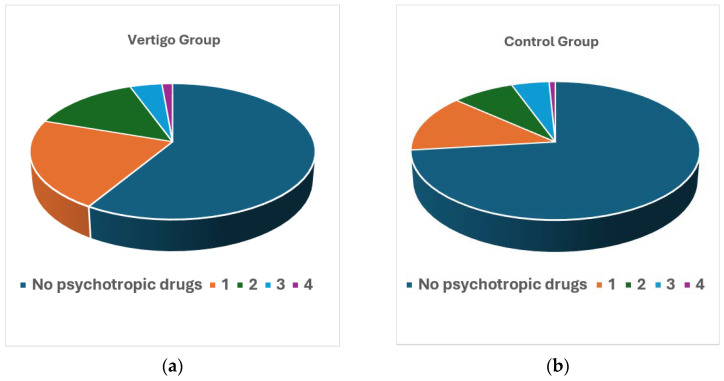
Number of different psychotropic drugs consumed in each group: (**a**) patients with vertigo; (**b**) control group.

The consumption of psychotropic drugs was studied in the group of patients with vertigo and in the control group. Table 2 and Figure 1 show the differences between both groups regarding the number of different psychotropic drugs simultaneously consumed (i.e., number of different types of medications that are usually taken, whether or not they belong to the same pharmacological group, and without considering the number of tablets). The consumption of psychotropic drugs was higher in the group of patients with vertigo compared to the controls, in all analyses carried out. Only 26.9% of the subjects in the control group consumed psychotropic drugs, while the percentage for patients with vertigo was 41.3% (Fisher’s exact test, *p* < 0.0001; OR = 1.914, CI95% (1.377; 2.662)). The mean number of psychotropic drugs consumed was also higher (Mann–Whitney test, *p* = 0.0003) in cases (0.68 ± 0.959) than in controls (0.47 ± 0.889). Considering that the maximum number of different drugs consumed is four, if this variable is analyzed as categorical, the difference continues to be statistically significant (Chi-square test, *p* = 0.041).

Table 3 shows the distribution of individuals observed in the vertigo and control groups according to the type of psychotropic drug consumed. The different combinations of psychotropic drugs consumed are also shown in detail.

When analyzing the consumption of each type of psychotropic drug (alone or in combination with another), this is higher in the group of patients with vertigo than in the control group, in all pharmacological groups studied, as can be seen in Table 4. This finding is especially relevant regarding “anxiolytics and hypnotics and sedatives” and “antidepressants” (alone, in combination with each other, or combined with other psychotropic drugs). It was found that 32.02% of patients with vertigo showed regular consumption of “anxiolytics and hypnotics and sedatives” alone or in association *versus* 20.55% of controls (Chi-square test, *p* = 0.001). Regarding antidepressants, 23.72% of patients with vertigo showed regular consumption of these psychotropic drugs alone or in association *versus* 16.60% of controls (Chi-square test, *p* = 0.0004). The differences observed in the consumption of “antiepileptics” and “antipsychotics” between both groups, although statistically significant, affect very few patients, so their clinical impact is limited.

No statistically significant differences in the consumption of psychotropic drugs between types of vestibular disorders were observed (Chi-square test, *p* = 0.740) (Table 5).

**Figure 2:**
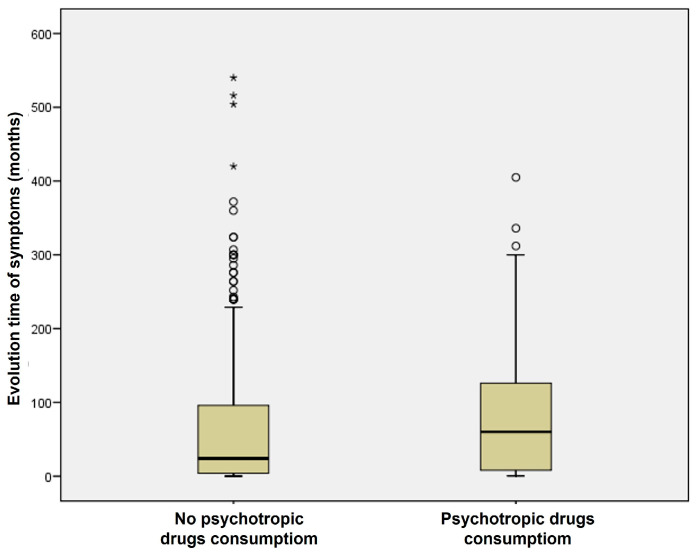
Consumption of psychotropic drugs and time evolution of vestibular symptoms in the group of patients with vertigo.

In the group of patients with vertigo, those who consumed psychotropic drugs showed a longer evolution time of their vestibular disorder (82.60 ± 84.125 months) than those who did not consume them (68.53 ± 98.634 months) (Figure 2). This difference was statistically significant (Mann–Whitney test, *p* = 0.002). Therefore, the longer the symptoms were present, the higher the prevalence of psychotropic drug use was observed.

## 4. Discussion

In our study, the percentage of patients with vertigo who consumed psychotropic drugs was higher than the percentage of the control group who did so. The mean number of psychotropic drugs consumed was also higher in cases than in controls. This higher consumption in the group of patients with vertigo was found for all pharmacological groups studied, being especially relevant regarding “anxiolytics and hypnotics and sedatives” and “antidepressants”. No statistically significant differences in the consumption of psychotropic drugs between types of vestibular disorders were observed. The longer the symptoms were present, the higher the prevalence of psychotropic drug use was observed.

The relationship between vertigo and psychological distress is a relevant issue that can worsen the effectiveness of treatment in patients who suffer from it. Vestibular symptoms, especially recurrent or chronic ones, frequently cause anxiety or depressive symptoms, due to the limitation they cause, and the feeling of vulnerability developed in patients. In fact, chronic anxiety and depressive symptoms are considered as triggers of persistent postural-perceptual dizziness (PPPD) [23], and patients may profit from different treatment strategies including pharmacological treatment with antidepressants (selective serotonin reuptake inhibitors (SSRIs) or serotonin–norepinephrine reuptake inhibitors (SNRIs)), vestibular rehabilitation, and cognitive behavioral therapy [24]. On the other hand, acute or long-term stress situations can trigger attacks of vertigo, and these vestibular symptoms can influence and promote anxiety and/or depression symptoms. Therefore, just as vertigo can occur with psychiatric symptoms, it is also true that these symptoms, especially anxiety and depression, can also contribute to vertigo. The influence of other environmental circumstances, such as diet [25,26], atmospheric pressure [27], or occupational exposure to noise and vibrations [28], on vestibular symptoms was studied and reported. The putative mechanisms underlying the relationship between vestibular symptoms and psychological distress (in particular, anxiety and/or depression) remain to be fully elucidated.

A recent study conducted by Wu et al. in 2024 found that anxiety and depression were not causes of MD, and vice versa, but neuroticism was proposed as the common cause of anxiety, depression, and MD [4]. Nevertheless, Subasi et al. (2023) reported a high correlation between MD and anxiety [5], and Lahiji et al. (2022) observed that the prevalence of anxiety and depression was higher in MD and BPPV patients than in healthy people, and MD had a higher effect on the incidence of anxiety and depression compared to BPPV [6]. In line with these results, Batinović et al. (2024) reported that anxiety and depression were more prevalent in patients with definite VM compared to patients with migraine without vertigo and healthy controls [3].

Our study analyzed the regular use of psychotropic drugs, excluding acute or unscheduled consumption, in order to avoid those psychotropic drugs prescribed for the treatment of acute vestibular syndrome. In our series, the consumption of psychotropic drugs was significantly higher in patients with vertigo than in the control group (patients with other otorhinolaryngological, non-vestibular symptoms). Subasi et al. (2023) concluded in their study that vertigo appears to be more intrusive than the other symptoms in MD patients [5]. In our study, more patients with vertigo consumed these drugs compared to controls, and the number of drugs consumed by these patients was greater than that in the control group. Taking into account the four groups of drugs analyzed (anxiolytics and related drugs, antidepressants, antiepileptics, and antipsychotics), this difference in consumption between cases and controls mainly affects the first two categories. Although the consumption of antiepileptics and antipsychotics is higher in patients with vertigo than in controls, it is very infrequent and does not allow for obtaining relevant clinical conclusions. High levels of prescription drug usage, including psychotropic medication, have been previously reported in elderly people with dizziness [7,8].

A relevant finding of our study is that the patient’s vestibular diagnosis does not seem to be related to the type of psychotropic drug consumed. A common hypothesis suggests that vestibular disorders, such as MD or VM, involving longer and more disabling vertigo attacks than BPPV, are more associated with psychological distress, and consequently, greater psychotropic drug consumption would be expected. However, in everyday practice, BPPV patients often express anxiety regarding head rotations that trigger vertigo. In our series, there are no differences between these three diagnostic groups. Although consumption is somewhat lower in patients with VN, which is a clinical picture with very intense vestibular symptoms, but limited in time, differences are not statistically significant. These findings agree with previous studies showing that the diagnosis of anxiety disorders was less frequent in patients with chronic vestibular disorders, such as bilateral vestibulopathy, than in those with episodic vertigo/dizziness [9].

However, in our series, there is a relationship between a longer duration of the disease and a higher consumption of psychotropic drugs. Therefore, it seems that the anxious or depressive clinical manifestations are a consequence of the repetition of the vestibular symptoms, rather than the duration and severity of each episode, and not the cause of them. Probably, even more limiting than the vertigo episodes themselves is the fear that they will appear suddenly and without warning. This fact can lead to anticipatory anxiety and interfere with daily activities including social and occupational ones. These limitations can lead to social withdrawal and exacerbate feelings of loneliness and isolation, having an impact on the perception of the patient’s quality of life [2]. Vestibular symptoms seem to be somewhat correlated with factors mediating anxiety or depression.

A strength of this study is the recording of psychotropic drug consumption as a method to quantify the mental health of patients. Most studies assess anxiety and depression with questionnaires [6]. These, although validated, are subjective, and their results can be influenced by many factors (e.g., mood on the day on which the questionnaire is answered, variability in the interpretation of the questions, mismatch between some of the questions and the socio-cultural variables of the individual). In our country, the prescription of psychotropic drugs requires prior evaluation by a physician and a confirmed diagnosis that justifies this pharmacological treatment. Therefore, we consider the proposed system to be more reliable than the use of questionnaires.

Our study has several limitations. Firstly, it is a single-center study, so the results obtained in our environment may not be extrapolated to other areas with different population characteristics. Secondly, to simplify the analysis, only the drugs consumed have been recorded, but not their doses. Recording the doses could allow us to better understand the severity of anxiety and depression symptoms, but it would have excessively fragmented the therapeutic groups. Thirdly, it could not be confirmed that the drug prescription had been provided by a psychiatrist. Finally, the pharmacological treatment effects were not analyzed in this study.

## 5. Conclusions

A relationship between vertigo and the consumption of psychotropic drugs was observed in our study. The consumption of psychotropic drugs was significantly higher in patients with vertigo than in the control group. The number of psychotropic drugs consumed was also greater in patients with vertigo. This difference in consumption was especially relevant in the group of anxiolytics, hypnotics, and sedatives and in the group of antidepressants. In patients with vertigo, there were no differences between diagnostic groups. The chronic course of vestibular disorder appears to be related to increased use of psychotropic drugs. Recording the consumption of these drugs is proposed as an objective method to better understand the psychological distress that patients with vertigo may suffer from.

## Figures and Tables

**Table 1:** Characteristics of participants included in the vertigo group.

Diagnosis	MD	VM	VN	BPPV
**Number of patients**	232	79	34	161
**Female/Male**	139/93	60/19	24/10	118/43
**Age (years)**	60.2 ± 12.149	45.7 ± 13.344	55.9 ± 14.649	63.9 ± 14.964
**Side**	Right: 98 Left: 81 Bilateral: 53		Right: 14 Left: 20	Right: 80 Left: 65 Bilateral: 16
**Canal**				Posterior: 123 Lateral: 18 —Canalithiasis: 6 —Cupulolithiasis: 12 Superior: 9 Multi-canal: 11
**Vestibular nerve**			Superior: 22 Inferior: 4 Both: 8	

**Table 2:** Regular consumption of psychotropic drugs and number of different psychotropic drugs consumed by cases and controls.

Number of Psychotropic Drugs Consumed	Patients with Vertigo (%)	Individuals of the Control Group (%)
1	21.54	13.44
2	14.23	7.91
3	4.15	4.74
4	1.38	0.79
**≥1**	**41.30**	**26.88**

**Table 3:** Distribution of subjects who consume different types of psychotropic drugs (alone or in association) in case and control groups.

Psychotropic Drugs	Patients with Vertigo (%)	Individuals of the Control Group (%)
Anxiolytics and hypnotics and sedatives (alone) (A + AA)	74 + 4 = 78 (15.42%)	21 A + 3 AA = 24 (9.48%)
Anxiolytics and hypnotics and sedatives (in association) (AB + AAB + ABB + A * + A ** + A ***)	54 + 9 + 9 + 2 + 3 + 7 = 84 (16.60%)	16 + 3 + 5 + 0 + 2 + 2 = 28 (11.07%)
**Anxiolytics and hypnotics and sedatives** **(alone or in association)**	**162 (32.02%)**	**52 (20.55%)**
Antidepressants (alone) (B + BB)	28 + 4 = 32 (6.32%)	12 + 0 = 12 (4.74%)
Antidepressants (in association) (AB + AAB + ABB + B * + B ** + B ***)	54 + 9 + 9 + 6 + 3 + 7 = 88 (17.39%)	16 + 3 + 5 + 1 + 3 + 2 = 30 (11.86%)
**Antidepressants (alone or in association)**	**120 (23.72%)**	**42 (16.60%)**
Anxiolytics and hypnotics and sedatives + antidepressants (alone) (AB + AAB + ABB + AABB + ABBB)	54 + 9 + 9 + 3 + 0 = 75 (14.82%)	16 + 3 + 5 + 1 + 1 = 26 (10.28%)
Anxiolytics and hypnotics and sedatives + antidepressants (in association) (AB * + AB **)	3 + 4 = 7 (1.38%)	1 + 0 = 1 (0.39%)
**Anxiolytics and hypnotics and sedatives + antidepressants (alone or in association)**	**82 (16.21%)**	**27 (10.67%)**
Antiepileptics (alone) (C + CC)	7 + 1 = 8 (1.58%)	1 + 0 = 1 (0.40%)
Antiepileptics (in association) (C * + C ** + C ***)	7 + 1+ 2 = 10 (1.98%)	0 + 2 + 0 = 2 (0.79%)
**Antiepileptics (alone or in association)**	**18 (3.56%)**	**3 (1.19%)**
**Antipsychotics (in association)** (D * + D ** + D ***)	3 + 2 + 2 = **7 (1.38%)**	1 + 2 + 0 = **3 (1.19%)**

**A**: anxiolytics and hypnotics and sedatives, **B**: antidepressants, **C**: antiepileptics, **D**: antipsychotics, * another psychotropic drug of those studied, ** two other psychotropic drugs of those studied, *** three other psychotropic drugs of those studied.

**Table 4:** Percentage of subjects who consume different types of psychotropic drugs (alone or in association) in case and control groups and statistical analysis of the differences between them (statistically significant if *p* < 0.05).

Psychotropic Drugs	Patients with Vertigo (%)	Individuals of the Control Group (%)	Chi-Square (p)
Anxiolytics and hypnotics and sedatives (alone)	15.42	9.48	0.0005
Anxiolytics and hypnotics and sedatives (alone or in association)	32.02	20.55	0.001
Antidepressants (alone)	6.32	4.74	0.0005
Antidepressants (alone or in association)	23.72	16.60	0.0004
Anxiolytics and hypnotics and sedatives + antidepressants (alone or in association)	16.21	10.67	0.001
Antiepileptics (alone or in association)	3.56	1.19	0.0003
Antipsychotics (in association)	1.38	1.19	0.0005

**Table 5:** Distribution of consumption of psychotropic drugs in patients with vertigo by diagnostic groups (Chi-square test, *p* = 0.740).

Consumption	MD (%)	VM (%)	VN (%)	BPPV (%)	Total
No psychotropic drug consumption	135 (58.19)	45 (56.96)	23 (67.65)	94 (58.39)	297
Psychotropic drug consumption	97 (41.81)	34 (43.04)	11 (32.35)	67 (41.61)	209
**Patients**	232	79	34	161	506

## Data Availability

The data presented in this study are available on request from the corresponding author due to privacy, legal, and ethical reasons.

## Abbreviations

The following abbreviations are used in this manuscript:

MD	Ménière’s disease
VM	Vestibular migraine
VN	Vestibular neuritis
BPPV	Benign paroxysmal positional vertigo
